# Isolated hypoglossal nerve palsy from internal carotid artery dissection related to *PKD*-1 gene mutation

**DOI:** 10.1186/s12883-019-1477-1

**Published:** 2019-11-08

**Authors:** Zhaoyao Chen, Jun Yuan, Hui Li, Cuiping Yuan, Kailin Yin, Sen Liang, Pengfei Li, Minghua Wu

**Affiliations:** 1 Department of Neurology, Jiangsu Province Hospital of Chinese Medicine, Affiliated Hospital of Nanjing University of Chinese Medicine, 155 Hanzhong Road, Nanjing, 210002 Jiangsu China; 2 Department of Radiology, Jiangsu Province Hospital of Chinese Medicine, Affiliated Hospital of Nanjing University of Chinese Medicine, Nanjing, 210002 Jiangsu China; 3 Department of Clinical Laboratory, Jiangsu Province Hospital of Chinese Medicine, Affiliated Hospital of Nanjing University of Chinese Medicine, Nanjing, 210002 Jiangsu China

**Keywords:** Hypoglossal nerve palsy, Internal carotid artery dissection, High-resolution MRI, Target genes capture and high-throughput sequencing, PKD1 gene mutation

## Abstract

**Background:**

Internal carotid artery dissection has been well recognized as a major cause of ischaemic stroke in young and middle-aged adults. However, internal carotid artery dissection induced hypoglossal nerve palsy has been seldom reported and may be difficult to diagnose in time for treatment; even angiography sometimes misses potential dissection, especially when obvious lumen geometry changing is absent.

**Case presentation:**

We report a 42-year-old man who presented with isolated hypoglossal nerve palsy. High-resolution MRI showed the aetiological dissected internal carotid artery. In addition, a potential genetic structural defect of the arterial wall was suggested due to an exon region mutation in the polycystic-kidney-disease type 1 gene.

**Conclusions:**

Hypoglossal nerve palsy is a rare manifestations of carotid dissection. High-resolution MRI may provide useful information about the vascular wall to assist in the diagnosis of dissection. High-throughput sequencing might be useful to identify potential cerebrovascular-related gene mutation, especially in young individuals with an undetermined aetiology.

## Background

Internal carotid artery dissection (ICAD) has been well recognized as a major cause of ischaemic stroke in young and middle-aged adults [1]. However, ICAD induced hypoglossal nerve palsy is involved in only 5% of cases [2]. Additionally, due to the combined sudden symptoms of lower cranial palsies and relative begin clinical features, hypoglossal nerve palsy due to ICAD may be difficult to diagnose in sufficient time for treatment, even angiography sometimes misses the potential dissection, especially when obvious changes in lumen geometry are absent.

Patients with spontaneous arterial dissection have been suggested to have a potential genetic structurale defects of the arterial wall. Heritable connective tissue diseases, such as Ehlers–Danlos syndrome type IV, Marfan’s syndrome, and autosomal dominant polycystic kidney disease (ADPKD), have been associated with an increased risk of spontaneous ICAD [1, 3].

## Case presentation

A 42-year-old male presented with slight right neck pain, slurred speech and slight difficulty in chewing. He was previously healthy. The patient noted right side neck pain and a slight headache upon waking one morning, and 2 days later, after a nap at his desk, he experienced slurred speech and slight difficulty in chewing. The patient then visited the clinic, but head CT and MRI indicated no obvious abnormalities. He was then referred to our department.

**Fig. 1:**
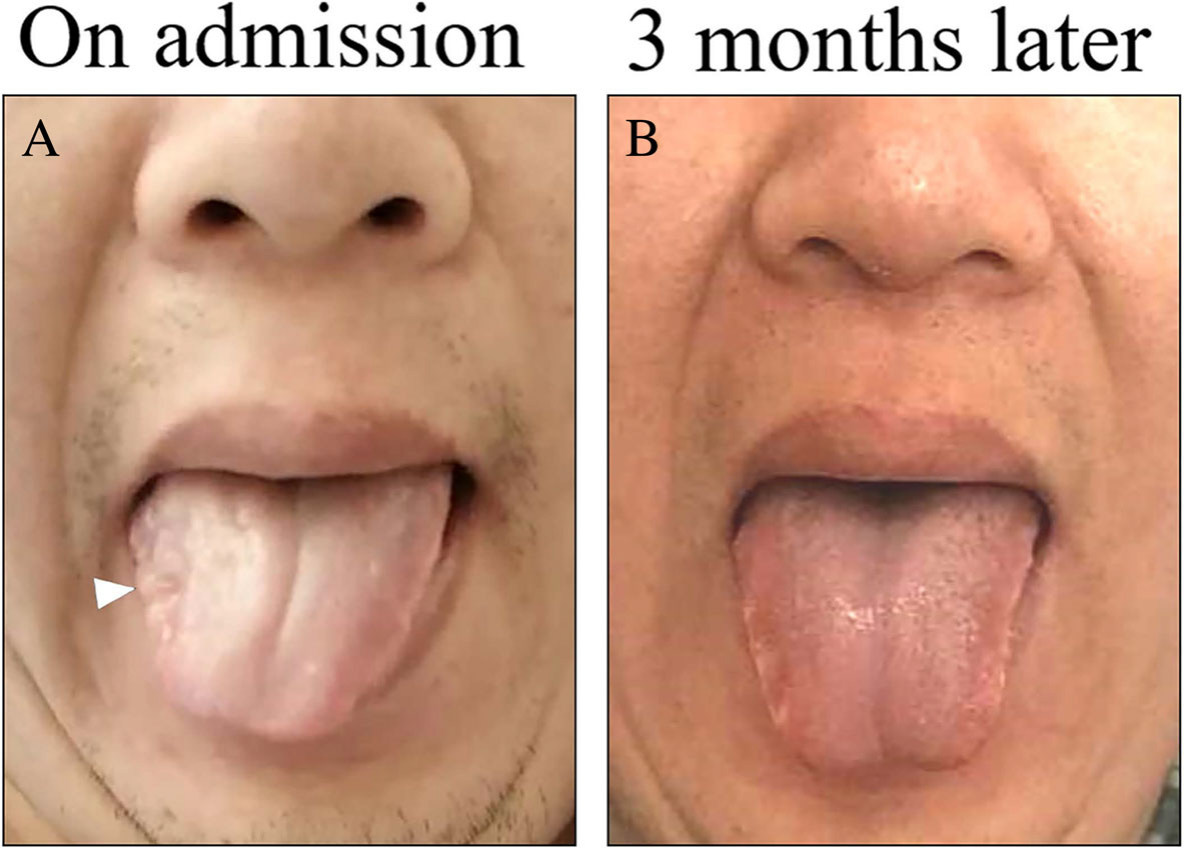
Clinical photography of hypoglossal nerve palsy. Clinical photography demonstrating (**a**) right side deviation when sticking out the tongue with a local palsy (white arrowhead) and (**b**) resolution 3 months later

On admission, a physical examination showed that the patient weighed 85-kg, exhibiting mild obesity and had a slight speech disturbance. His cardiopulmonary and otorhinolaryngologic systems were normal. Neurological examination only revealed a right deviation of a protruded tongue with local palsy (Fig. 1a).

**Fig. 2:**
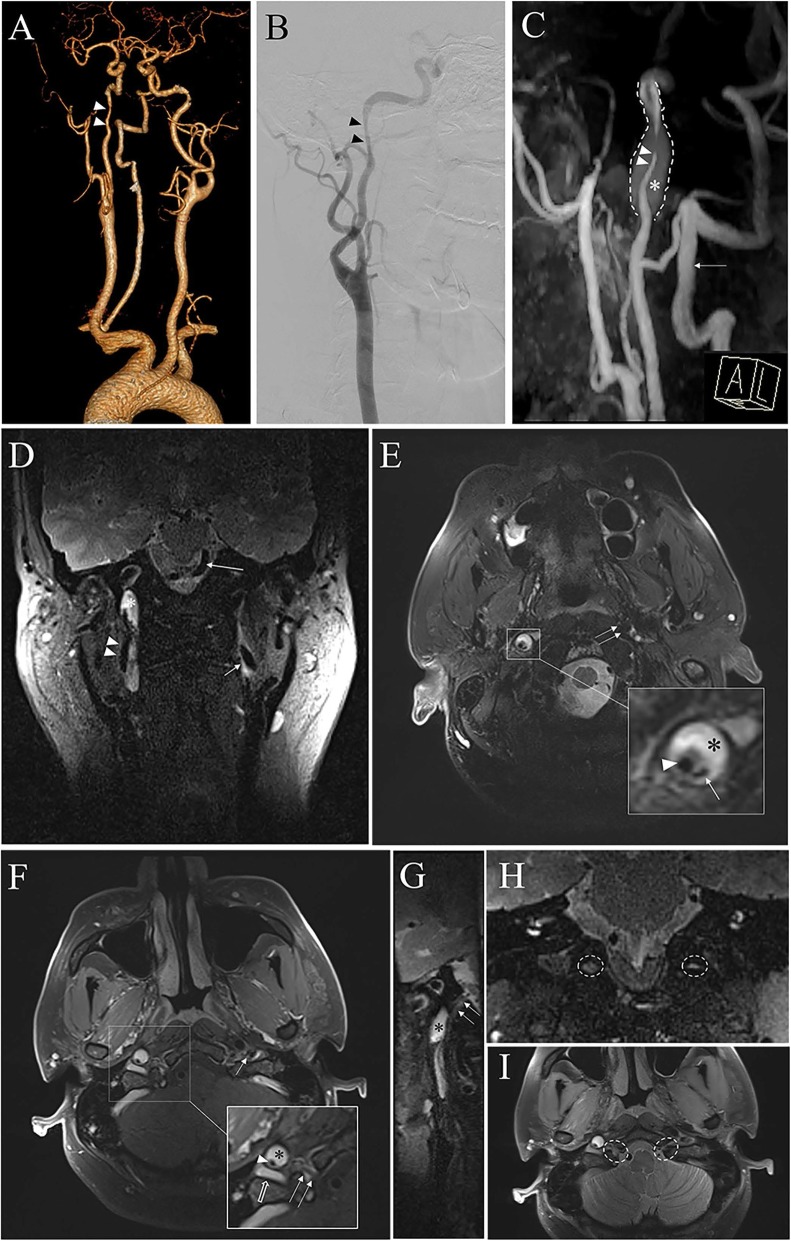
Imaging examination of dissection. **a** CTA and (**b**) DSA showing only the stenosis (white arrowhead) of right ICA, about 3.5 cm above the carotid bulb and 2.5 cm in length, the (**c**) MRA showing the right ICA stenosis as well as the surrounding hematoma (white asterisk) within the arterial wall. The white arrow indicates the right vertebral artery. **d** Coronal T2-tse-vfl sequence showing the hypo signal of right ICA and hyper signal hematoma (white asterisk), tortuous right vertebrobasilar artery (long white arrow) cross the midline to the left, and normal left ICA (short white arrow). **e** Axial T2-tse-tra-fs sequence showing enlarged right ICA with hematoma within vascular wall (black asterisk), as well as a stenotic true lumen (white arrowhead), and the opening of the false lumen (single white arrow) which may indicate the tearing of intima. Normal left ICA was annotated with a double white arrow. **f** Axial T1-tse-tra-2 mm sequence and (**g**) sagittal MPR showing the dissected right ICA, certificate with stenotic true lumen (white arrowhead) and within vascular crescent hematoma (black asterisk); the deformed internal jugular vein (white blank arrow) and hypoglossal nerve within hypoglossal canal (double white arrow) may be compressed by enlarged right ICA. **h** coronal T2-tse-vfl sequence and (**i**) pd-tse-tra-fs sequence showing the contents within the right hypoglossal canal has a fatter shape larger than that within the left (white dotted oval)

Routine blood test results were normal. However, a suspected diagnosis of right ICA dissection was suggested based on the clinical history and demonstration of obvious segmental narrowing with CTA and DSA (Fig. 2a and b). The diagnosis was finally confirmed by a high-resolution MRI (HRMRI) scan of the responsible segment of the ICA, which showed considerable segmental narrowing with an enlarged artery lumen, combined with a “double cavity”, intima tear, and haematoma within the vascular wall (Fig. 2c, d and e). We also found a tortuous right basilar artery (Fig. 2d) cross the midline to the left. T1-fat-suppression scan showed that the hypoglossal nerve was closely adjacent to the dilated internal carotid artery (Fig. 2f and g). Interestingly, the perineural structure within the right hypoglossal canal seemed larger than that on the left side (Fig. 2h and i), and a compressed, deformed internal jugular vein was observed (Fig. 2f).

**Fig. 3:**
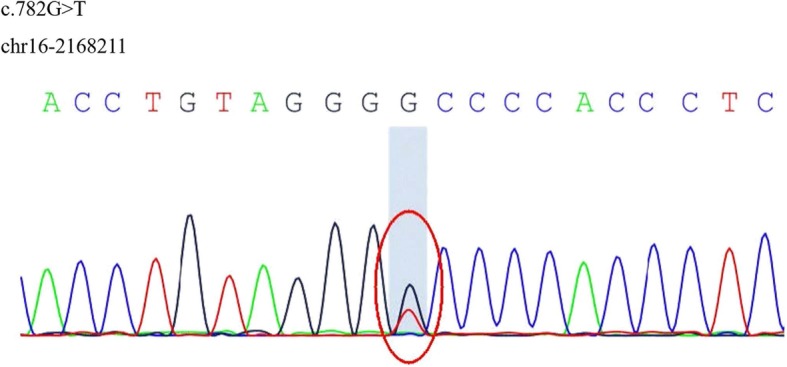
Target-genes capture and high-throughput sequencing. A heterozygous mutation of the polycystic kidney disease type 1 (PKD1) gene which located in the exon region of this gene: c.782G > T (guanine > thymidine)

Target-genes-capture and high-throughput-sequencing showed that the patient had a heterozygous mutation of the polycystic kidney disease type 1 (*PKD*-1) gene, which was located in the exon region of this gene: c.782G > T (guanine > thymidine), resulting in an amino acid change p.G261 V (glycine > valine) (Fig. 3). The sample indicated two other heterozygous mutations in von Willebrand disease (VWF) related genes. Multiple small liver cysts were found on an abdominal CT scan, however, the patient did not present polycystic kidney disease (Additional file 1: Figure S1).

The patient received antiplatelet therapy with clopidogrel, and his clinical condition gradually improve. After 3 months of follow-up, the tongue paresis had fully recovered (Fig. 1b). A CTA examination was performed 6 months later which showed significant recovery of the internal carotid lumen, with only mild residual stenosis (Additional file 2: Figure S2).

## Discussion and conclusions

ICAD induced hypoglossal nerve palsy is rare, two mechanisms with the consensus are as follows, first, mechanical compression or stretching of the nerve by the expanded dissected arterial wall with the sub-adventitial hematoma [2]. Second, nerve ischaemia caused by compromised blood supply [2]. In our case, compression of the hypoglossal nerve was primarily suspected, as HRMRI demonstrated that the nerve passed immediately beneath the dissected ICA, and that the internal jugular vein was compressed (Fig. 2f and g). Interestingly, the enlarged structure within the hypoglossal canal on the affected side (Fig. 2h and i) may due to suspicious venous reflux obstruction to the internal jugular vein as suggested by a previous study [4], which may also indicate the compression mechanism. The hypoglossal canal contains the canalicular segment of the 12th cranial nerve, a branch of the ascending pharyngeal artery, and the anterior condylar venous plexus [5]. Previous studies suggested that an enlarged canalicular venous plexus may be responsible for the hypoglossal nerve palsy [5, 6]. Dynamic contrast-enhanced MRI could be considered to compare the differences in enhancement timing of hypoglossal canal structures (arteries, veins, and dura) for confirmation [7].

A previous study suggested that ICAD patients with local signs were associated with a benign clinical course and a favourable outcome [8]. However, this condition requires treatment due to the subsequent risk of ischaemic stroke [9], especially within the first 2 weeks [10]. Furthermore, there is no consensus regarding the optimal therapeutic regimen for spontaneous ICAD. A recent meta-analysis find no differences regarding antiplatelate or anticoagulant in stroke prevention of cervical or vertebral artery dissection patients [11].

In our case, a heterozygous mutation in the exon region of the *PKD*-1 gene was detected which could theoretically cause the disease. Previous studies have suggested that ICAD patients could have a constitutional, at least to some extent, genetically determined weakness of the vessel wall. More than half of patients with carotid artery dissection were found to have skin connective tissue abnormalities, including composite fibrils within mid-dermal collagen bundles and enlarged fibrils [12]. A previous study also found that concomitant arterial abnormalities such as tortuosity, kinking or coiling ICA was common [13], and our patient also had a tortuous basilar artery. In addition, heritable connective tissue disorders such as Ehlers–Danlos syndrome, Marfan’s syndrome, are associated with an increased risk of spontaneous ICAD [3]. ADPKD has also been rarely described as correlating with dissection of the cerebral arteries [14–16]. ADPKD is the most common inherited renal cystic disease and it is associated with various extrarenal manifestations, such as polycystic liver disease [17], cardiac valvular anomalies, and colonic diverticular and vascular complications. The prevalence of intracerebral aneurysms in patients with ADPKD is 8–10% and it is therefore more common than in the general population [1]. Almost 90% of ADPKD cases have been attributed to mutations of the *PKD*-1 or *PKD*-2 genes [18]. These two genes encode polycystin, a membrane glycoprotein, located in arterial smooth muscle, and deficiency of this protein may play an important pathogenic role in arterial complications [19]. However, this patient did not present polycystic kidney disease, aside from the presence of multi-small-liver cysts. A previous study found that liver involvement is the most frequent extrarenal manifestation of ADPKD [20], and the disease is linked either to the *PKD-*1 or *PKD-*2 gene. We propose that, in this case, the *PKD-*1 gene mutation might be the underlying mechanism of the arterial wall weakness.

In conclusion, carotid dissection, as well as potential gene mutation, should be considered in young patients with isolated hypoglossal nerve palsy.

## Supplementary information

**Additional file 1: Figure S1.** Multi small liver cysts in upper abdomen CT. (A, B and C) CT scan found low density multi small liver cysts (white arrow), however, the (D) cyst was not detected in bilateral kidneys.

**Additional file 2: Figure S2.** Internal carotid artery stenosis follow-up by CTA. The CTA source images showed the severe stenosis (A and B, white arrow head) and vascular wall hematoma (A) on admission; We arranged a CTA follow-up 6 months later, the stenosis was compeletely resolved (C and D) and the hematoma was mostly absorbed.

## Data Availability

All data are available without restriction from the corresponding author on reasonable request.

## Abbreviations

ADPKD: Autosomal dominant polycystic kidney disease
CTA: CT angiography
DSA: Digital subtraction angiography
HRMRI: High-resolution MRI
ICA: Internal carotid artery
ICAD: Internal carotid artery dissection
*PKD*-1: Polycystic kidney disease type 1
*PKD*-2: Polycystic kidney disease type 2
VWF: Von Willebrand disease
