# Supplementary figures and images for: Isolated hypoglossal nerve palsy from internal carotid artery dissection related to PKD-1 gene mutation
Source: BMC Neurol. 2019 Nov 8;19:276. doi: 10.1186/s12883-019-1477-1 (PMC6839245; doi:10.1186/s12883-019-1477-1)

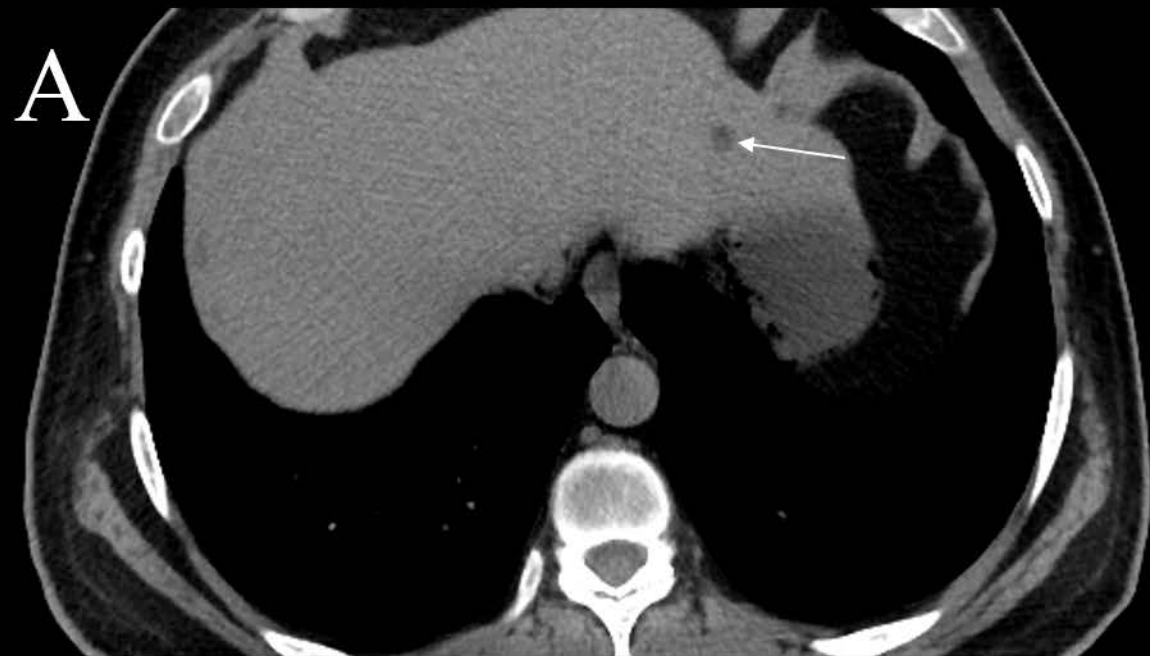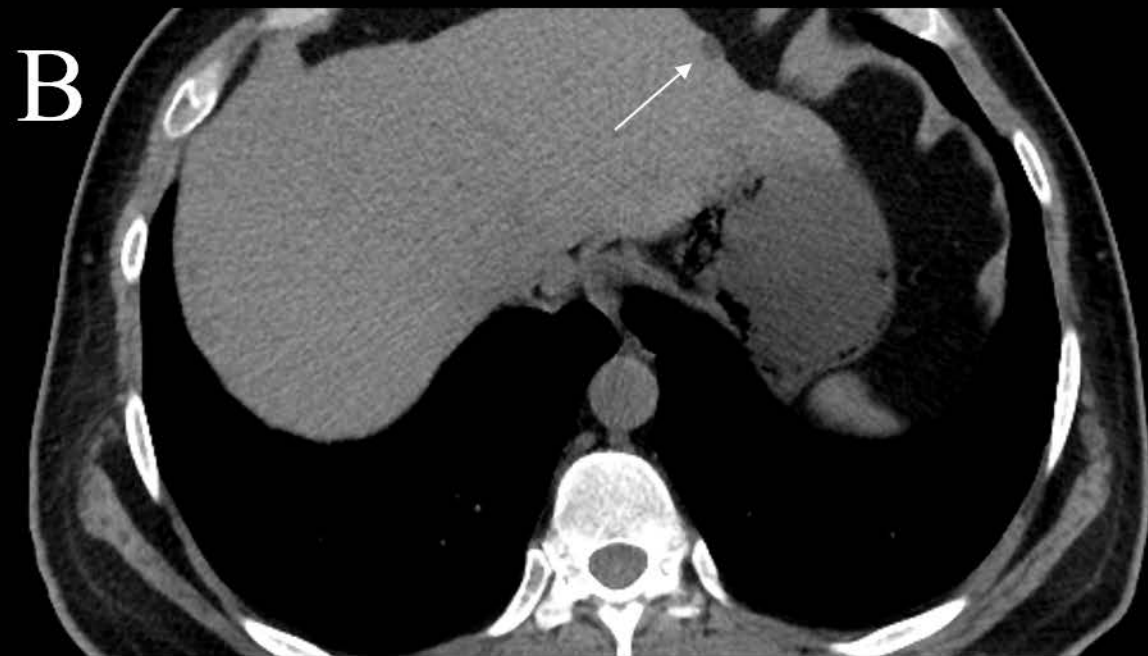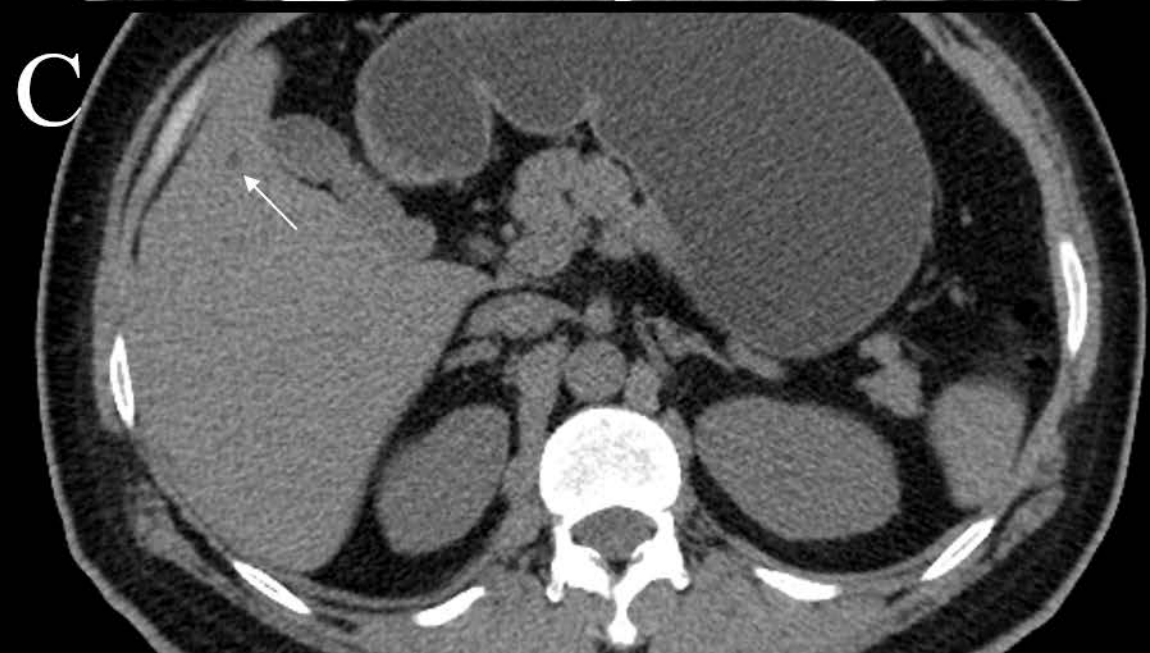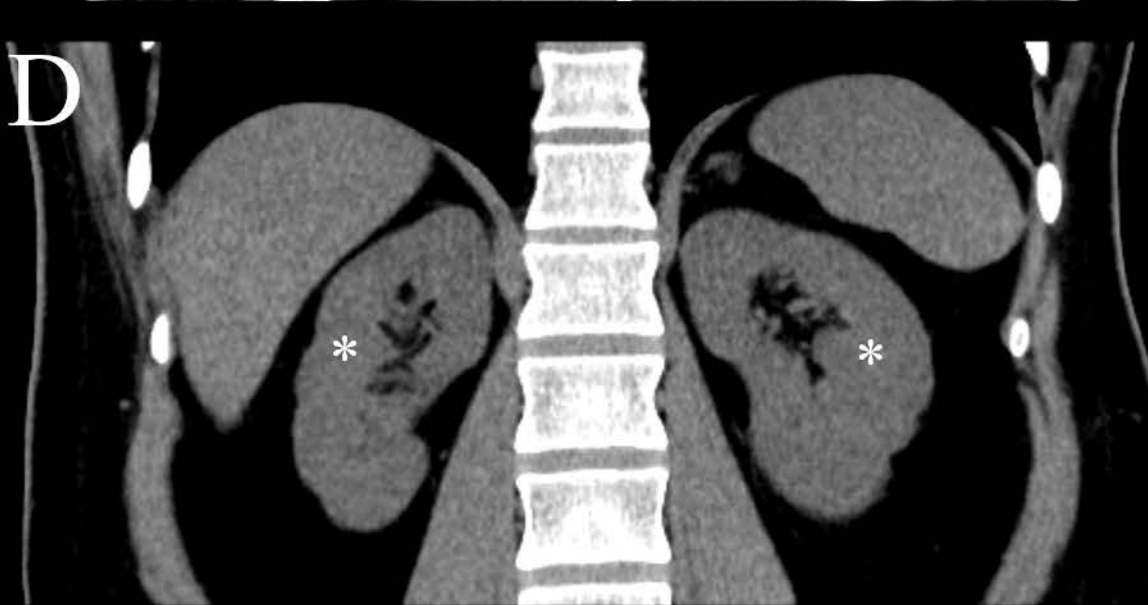

Supplement: Supplementary file 1 — Additional file 1: Figure S1. Multi small liver cysts in upper abdomen CT. (A, B and C) CT scan found low density multi small liver cysts (white arrow), however, the (D) cyst was not detected in bilateral kidneys. [file 12883_2019_1477_MOESM1_ESM.pdf]

On admission

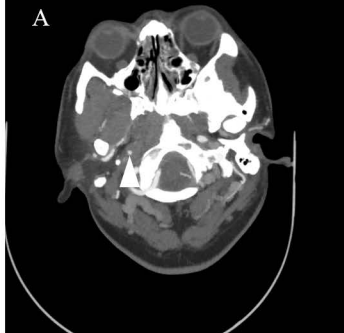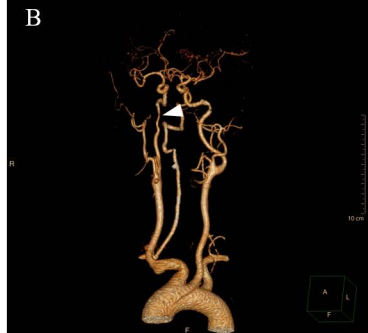

6 months later

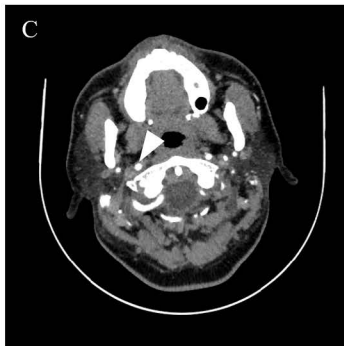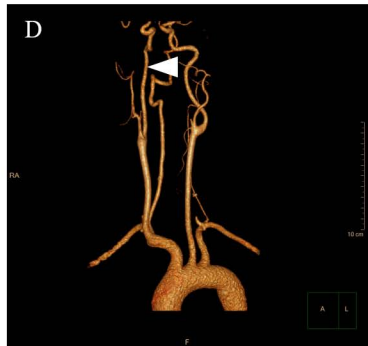

Supplement: Supplementary file 2 — Additional file 2: Figure S2. Internal carotid artery stenosis follow-up by CTA. The CTA source images showed the severe stenosis (A and B, white arrow head) and vascular wall hematoma (A) on admission; We arranged a CTA follow-up 6 months later, the stenosis was compeletely resolved (C and D) and the hematoma was mostly absorbed. [file 12883_2019_1477_MOESM2_ESM.pdf]
